# Comparing and Contrasting the Cognitive Effects of Hippocampal and Ventromedial Prefrontal Cortex Damage: A Review of Human Lesion Studies

**DOI:** 10.1016/j.neuroscience.2017.07.066

**Published:** 2018-03-15

**Authors:** Cornelia McCormick, Elisa Ciaramelli, Flavia De Luca, Eleanor A. Maguire

**Affiliations:** a Wellcome Trust Centre for Neuroimaging, Institute of Neurology, University College London, 12 Queen Square, London WC1N 3BG, UK; b Dipartimento di Psicologia, Università di Bologna, Bologna, Italy; c Centro studi e ricerche di Neuroscienze Cognitive, Cesena, Italy

**Keywords:** vmPFC, hippocampus, amnesia, autobiographical memory, scene construction, decision making

## Abstract

The hippocampus and ventromedial prefrontal cortex (vmPFC) are closely connected brain regions whose functions are still debated. In order to offer a fresh perspective on understanding the contributions of these two brain regions to cognition, in this review we considered cognitive tasks that usually elicit deficits in hippocampal-damaged patients (e.g., autobiographical memory retrieval), and examined the performance of vmPFC-lesioned patients on these tasks. We then took cognitive tasks where performance is typically compromised following vmPFC damage (e.g., decision making), and looked at how these are affected by hippocampal lesions. Three salient motifs emerged. First, there are surprising gaps in our knowledge about how hippocampal and vmPFC patients perform on tasks typically associated with the other group. Second, while hippocampal or vmPFC damage seems to adversely affect performance on so-called hippocampal tasks, the performance of hippocampal and vmPFC patients clearly diverges on classic vmPFC tasks. Third, although performance appears analogous on hippocampal tasks, on closer inspection, there are significant disparities between hippocampal and vmPFC patients. Based on these findings, we suggest a tentative hierarchical model to explain the functions of the hippocampus and vmPFC. We propose that the vmPFC initiates the construction of mental scenes by coordinating the curation of relevant elements from neocortical areas, which are then funneled into the hippocampus to build a scene. The vmPFC then engages in iterative re-initiation via feedback loops with neocortex and hippocampus to facilitate the flow and integration of the multiple scenes that comprise the coherent unfolding of an extended mental event.

## Highlights

• The vmPFC and hippocampus are closely connected brain regions whose functions are still debated.
• Here we directly compared the cognitive changes in humans with either bilateral hippocampal or bilateral vmPFC damage.
• Hippocampal and vmPFC damage both affect classic ‘hippocampal’ tasks such as autobiographical memory recall.
• Hippocampal and vmPFC damage have opposite effects on classic ‘vmPFC’ tasks such as moral decision making.
• We propose a hierarchical network model where vmPFC initiates mental imagery including hippocampal scene construction.

## Introduction

Human neuroimaging has consistently revealed co-activation of the hippocampus and the ventromedial prefrontal cortex (vmPFC), and strong connectivity between them (Andrews-Hanna et al., 2014, Catani et al., 2013, Spreng et al., 2009, Eichenbaum, 2017). The hippocampus is typically associated with episodic or autobiographical memory retrieval of personal past experiences (Addis et al., 2007b, Svoboda et al., 2006), and these functions are impaired when the hippocampi are damaged (Scoville and Milner, 1957, Steinvorth et al., 2005, Squire, 1992). In contrast, the vmPFC is typically linked with decision-making, emotion and social abilities, and lesions to this area compromise these functions (Bechara, 2004, Delgado et al., 2016, Fellows, 2011). In recent years, however, the idea that the hippocampus is exclusively mnemonic has been challenged, as neuroimaging and neuropsychological evidence accumulates to show that it might be involved in a much broader range of cognitive processes including scene construction (Hassabis et al., 2007, McCormick et al., 2017, Mullally et al., 2012), future thinking (Hassabis et al., 2007, Klein and Loftus, 2002, Kurczek et al., 2015), visual perception (Lee et al., 2005a) and decision making (McCormick et al., 2016). In a similar vein, damage to the vmPFC has been found to impair the ability to retrieve vivid autobiographical memories (Bertossi et al., 2016) and imagine scenes (Bertossi et al., 2015, Bertossi et al., 2017), drawing parallels with hippocampal-damaged patients. While hippocampal and vmPFC patients seem to have some deficits in common, nevertheless, the two patient types behave very differently and they diverge significantly in terms of other cognitive sequelae.

**Fig. 1:**
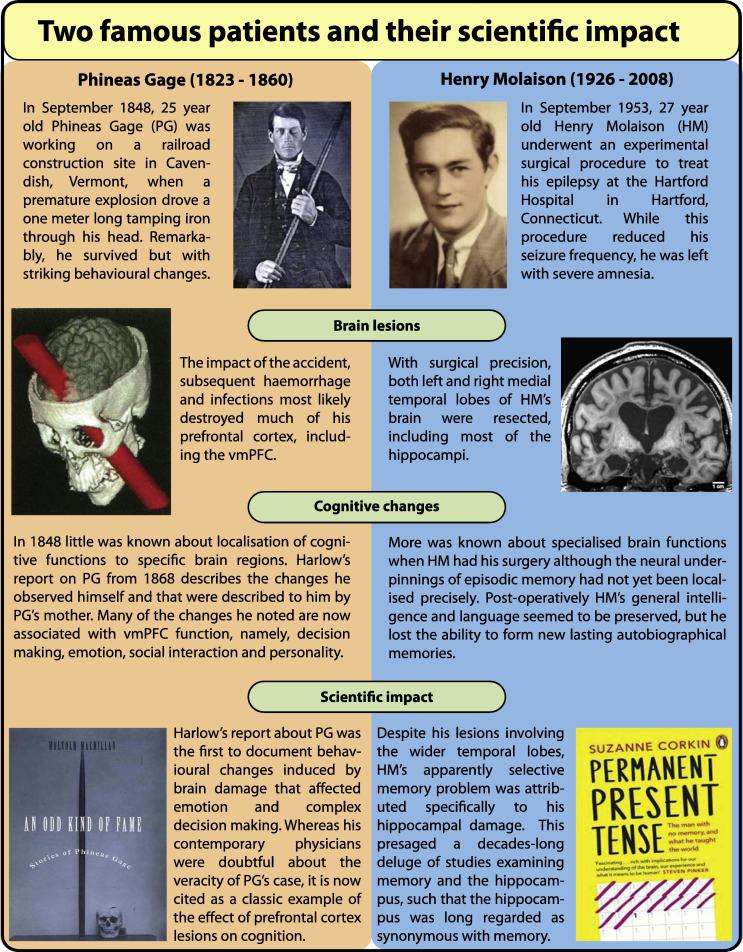
The lives and scientific impact of Henry Molaison and Phineas Gage. Images are used with permission from Corkin (2014) reprinted courtesy of Penguin Books, Augustinack et al. (2014), Damasio et al. (1994), Macmillan (2000) reprinted courtesy of The MIT Press and https://en.wikipedia.org/wiki/Phineas_Gage, and https://en.wikipedia.org/wiki/Henry_Molaison.

Given the lack of consensus about what these two brain areas do, here we take a different approach to considering this issue. Our premise is that directly comparing and contrasting the cognitive changes in individuals with either bilateral hippocampal damage or bilateral vmPFC damage may offer a new perspective on the contributions of these two brain regions to cognition. In fact, two of the most famous cases in the history of neuropsychology concern our regions of interest, namely, Henry Molaison (‘HM’) whose medial temporal lobes (and hippocampi) were surgically resected in an attempt to cure his epilepsy in 1953 (Corkin, 2002, Scoville and Milner, 1957), and Phineas Gage who sustained prefrontal cortex damage from a penetrating head injury in 1848 (Harlow, 1848, Harlow, 1869). Since their scientific impact still resonates today (Annese et al., 2014, Corkin, 2002, Corkin, 2014, Corkin et al., 1997, Damasio et al., 1994, Dossani et al., 2015, Harlow, 1848, Harlow, 1869, Macmillan, 2000, Ratiu et al., 2004, Scoville and Milner, 1957, Thiebaut de Schotten et al., 2015, Van Horn et al., 2012), their brain lesions and cognitive changes are described in Fig. 1.

We first consider cognitive functions that are usually impaired in patients with hippocampal damage, such as autobiographical memory retrieval, and then examine the performance of patients with vmPFC lesions on these tasks. We then take cognitive functions that are typically compromised following vmPFC damage, such as decision making, and look at how these are affected by hippocampal damage. We acknowledge that these functions are supported by many brain regions and not only the hippocampus or vmPFC. Nevertheless, this approach enables us to curate and consider a large literature within a clear, albeit simple, structure. We also recognize that there is much in the vast hippocampal and vmPFC literature that we do not cover, not least animal studies (see Eichenbaum, 2017) and human functional neuroimaging work. Our prime focus here is on making comparisons between hippocampal and vmPFC patients, and consequently our emphasis is on topics and tasks where there is neuropsychological evidence from both groups. Toward the end of the review we set out some preliminary ideas about what the hippocampus and vmPFC might contribute in common and differentially to our mental life, and suggest future directions that we think are important to pursue.

## Anatomy and connectivity

### Anatomy of the hippocampus and vmPFC

**Fig. 2:**
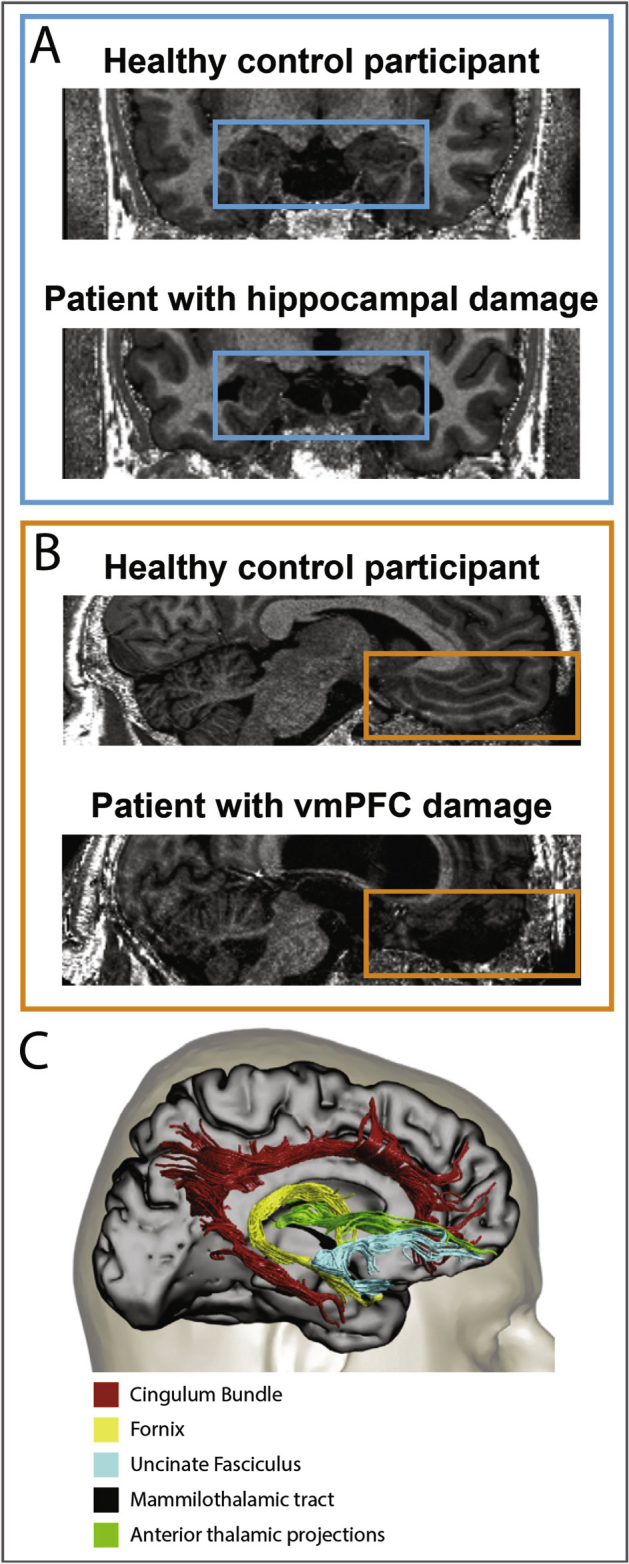
Anatomical location, connectivity and examples of lesions to the hippocampus and vmPFC. A. Structural MR coronal images from an example patient with selective bilateral hippocampal damage and an age-, gender- and IQ-matched healthy control participant. Images are displayed in native space corresponding approximately to the position of y = −10 in the MNI coordinate system. B. Structural MR sagittal images from an example patient with bilateral vmPFC damage and an age-, gender- and IQ-matched healthy control participant. Images are displayed in native space corresponding approximately to the position of x = 2 in the MNI coordinate system. C. An overview of the main anatomical connections between the hippocampi and the vmPFC using diffusion-weighted imaging in humans. The anatomical images of the healthy control and patients with hippocampal and vmPFC damage were acquired in accordance with the approval of the local ethics committee at our Centre. The connectivity image is adapted with permission from Catani et al. (2013).

The hippocampus is located in the medial temporal lobe (MTL; Fig. 2) of each hemisphere and has a distinct, curved shape that has been likened to the appearance of a seahorse (Amaral and Witter, 1989). It consists of two layers that are tightly rolled up inside each other. The first of these, the dentate gyrus, is wrapped around the second layer, forming a semicircle in cross-sectional views. The second layer consists of a series of Cornu Ammonis (CA) areas that define the subfields of the hippocampus namely, CA1, CA2, CA3, and CA4 (Sloviter and Lomo, 2012). Other parts of the hippocampus include the subiculum, presubiculum, parasubiculum, prosubiculum and the uncus (Witter, 1993). Along with the hippocampus, the neighboring entorhinal, perirhinal and parahippocampal cortices define the MTL. In this MTL system, the entorhinal cortex is the main gateway between most neocortical brain regions and the hippocampus. Recent developments in high-resolution (f)MRI have made it possible to delineate specific subregions within the human hippocampus on MRI scans (Dalton et al., 2017) and this is starting to illuminate their functional contributions (Zeidman et al., 2015a).

The vmPFC is a part of the prefrontal cortex (PFC) in mammals, comprising its bottom (ventral) and central (medial) portions (Mackey and Petrides, 2009, Mackey and Petrides, 2010, Ongur et al., 2003, Ongur and Price, 2000); Fig. 2). While there are no clear anatomical landmarks for this area, it is generally defined as the subgenual region, namely, beneath the genu of the corpus callosum. The vmPFC includes Brodmann areas 10, 14, 25, and 32, as well as portions of Brodmann areas 11, 12, and 13. It is surrounded by other parts of the prefrontal cortex that are commonly described based on their locations as ventrolateral, dorsomedial and dorsolateral PFC, and these are connected via short frontal pathways (Catani et al., 2012).

The main focus of this review is on individuals with damage that primarily involves either the hippocampus or vmPFC bilaterally (Fig. 2). However, where we feel it is pertinent to the discussion, we also consider neuropsychological studies where the damage was somewhat wider. In a similar vein, some older, pre-MRI neuropsychological studies that contrasted patients with bilateral MTL damage to patients with frontal lobe lesions offer valuable insights for the current review. We acknowledge that lesion characterizations in these patients by necessity lacked precision and should be appropriately caveated. A further note concerns vmPFC damage, which commonly results from a ruptured aneurysm and where treatment often involves insertion of a metal clip to stop the bleeding. This can preclude MRI scanning, which imposes constraints on evaluating lesions in detail and on the assessment of whether connectivity has been compromised.

### Connectivity between the hippocampus and vmPFC

In humans, the hippocampus and vmPFC are anatomically connected via three main reciprocal connections - the uncinate fasciculus, the fornix and the cingulum bundle (Catani et al., 2013, Concha et al., 2005, Malykhin et al., 2008). A fourth indirect pathway connects the vmPFC to the hippocampus via the mammillothalamic tract and anterior thalamic projections. The uncinate fasciculus connects the anterior part of the temporal lobe, including hippocampus, to the ventral and polar areas of the frontal cortex. Fibers of the fornix arise mainly from the hippocampus and entorhinal cortex, and connect the two hippocampi to each other and to the mammillary bodies. In addition, fornix fibers travel forward beneath the corpus callosum to the most posterior part of the vmPFC. The cingulum bundle is a large pathway containing fibers of different lengths, with the longest fibers connecting the anterior hippocampus and parahippocampal gyrus to the vmPFC. These fibers run above the corpus callosum with shorter fibers joining and leaving the cingulum bundle along its length. Overall, it seems that the anterior hippocampus has particularly strong connections with the vmPFC, and this has been confirmed by findings from diffusion-weighted imaging and functional connectivity of resting state fMRI data (Adnan et al., 2016, Andrews-Hanna et al., 2010).

Lesions to either the hippocampus or vmPFC likely impact their connectivity and, as these disconnections are usually messy (e.g., following a ruptured aneurysm), they can be partial or total, and might only affect one, two or all three of the main pathways connecting the hippocampus and vmPFC (Fig. 2; Hepdurgun et al., 2016, Liao et al., 2011). This disconnection makes it problematic to isolate the independent functions of both regions. Moreover, anatomical routes that connect the hippocampus and vmPFC to other parts of the brain can also be disrupted. These other brain areas are potentially transfer stations, serving as indirect anatomical connections between hippocampus and vmPFC. A prominent example of this is the thalamus, which receives direct input from both structures (Fig. 2; Catani et al., 2013).

Given that lesion extent and connectivity can be difficult to establish, one might wonder whether it is worth testing patients at all, as it might seem impossible to draw any clear-cut conclusions about the specific contributions of a brain region. However, individuals with damage to distinct brain areas can behave differently. Furthermore, while any one study alone might not be completely conclusive, by reviewing the literature we believe that significant motifs are evident that may be helpful in understanding the common and differential contributions of the hippocampus and vmPFC to cognition.

## Functions typically linked with the hippocampus

In her book about HM’s life, Corkin wrote that one of the first things that was noticed after HM woke up from the surgical removal of his bilateral MTLs was his inability to memorize his caregivers who attended to him several times a day (Corkin, 2014). In addition, he failed to learn the day-to-day routines of the hospital and the route to his bathroom (Corkin, 2014). By the time HM left the hospital two weeks after surgery, it was clear he had lost the ability to form new lasting autobiographical memories. It then gradually became evident that other cognitive functions depend on hippocampal integrity. For example, O’Keefe and colleagues discovered that the hippocampus in rodents plays a key role in spatial navigation (O'Keefe and Dostrovsky, 1971, O'Keefe and Nadel, 1978, O'Keefe and Speakman, 1987), a finding that has now been extensively replicated in humans (Maguire et al., 1998, Maguire et al., 2000, Maguire et al., 2006, Rosenbaum et al., 2000, Schinazi et al., 2013, Wolbers et al., 2007, Spiers and Maguire, 2006, Woollett and Maguire, 2011). In the last decade in particular, the hippocampus has been implicated in supporting even more functions (Koelsch, 2014, Lee et al., 2005b, Maguire and Mullally, 2013). It is impossible to consider here all of the instances where hippocampal involvement has been reported in a cognitive task. Instead, we sample across a range of functions chosen because there are data from both hippocampal and vmPFC patients which, surprisingly, is not that common. We briefly reprise how individuals with bilateral hippocampal damage do on tasks tapping these functions, before examining the performance of patients with vmPFC lesions.

### Autobiographical memory

#### Patients with hippocampal damage

The ability to form and retrieve detailed autobiographical memories is the function most associated with the hippocampus and the hallmark of hippocampal amnesia. Since the first observations of the recovering HM, the neuropsychological profile of hippocampal amnesia has been refined. For example, at first it seemed that individuals with bilateral MTL damage including the hippocampi were only impaired in laying down traces for new autobiographical memories but were able to recall remote autobiographical memories (Scoville and Milner, 1957, Squire, 1992). However, it is now widely accepted that recalling remote and recent autobiographical memories is impaired following bilateral hippocampal damage if the task requires retrieval that is vivid and detailed (Addis et al., 2007a, Rosenbaum et al., 2008, Rosenbaum et al., 2009, St-Laurent et al., 2009, St-Laurent et al., 2014, St-Laurent et al., 2011, Steinvorth et al., 2005, Viskontas et al., 2000; but see Dede et al., 2016, Kirwan et al., 2008, Squire et al., 2010 for an alternative view that the hippocampus is not critical for retrieval of remote autobiographical memories). In contrast, patients with bilateral hippocampal damage remember facts about their lives such as their home address and the name of their school (Nadel and Moscovitch, 1997, Winocur and Moscovitch, 2011). What they lack in their autobiographical recollection is episodic details, that is, a clear picture in their mind’s eye of any particular moment, or scene, from a past event (Kurczek et al., 2015, Rosenbaum et al., 2008, Steinvorth et al., 2005). This impairment is clear in reports of autobiographical memories, but also seems to be true for more generic, script-like events, such as going grocery shopping, presumably calling on the same processes of visualizing mental scenes (St-Laurent et al., 2009).

Only a small number of studies have scanned patients with hippocampal damage using fMRI while they were attempting to recall autobiographical memories (Addis et al., 2007a, Berryhill et al., 2010, Maguire et al., 2001, St-Laurent et al., 2014). As predicted by their lesion site, the MTL typically showed decreased activation compared to controls. However, interestingly, in cases where patients had gist-like recollection which lacked vividness, this seemed to be associated with up-regulation of neocortical structures, in particular the vmPFC (Addis et al., 2007a, Maguire et al., 2001). The same trade-off between hippocampal and vmPFC activation during autobiographical memory retrieval was found in a longitudinal case study examining a patient with semantic dementia (Maguire et al., 2010). Early in the disease process memory retrieval was still vivid and intact, and the authors reported hippocampal activation. However, a year later, when autobiographical memories lost recollective qualities, vmPFC activation was elevated. This begs the question as to what the vmPFC might be contributing to autobiographical memory.

#### Patients with vmPFC damage

**Fig. 3:**
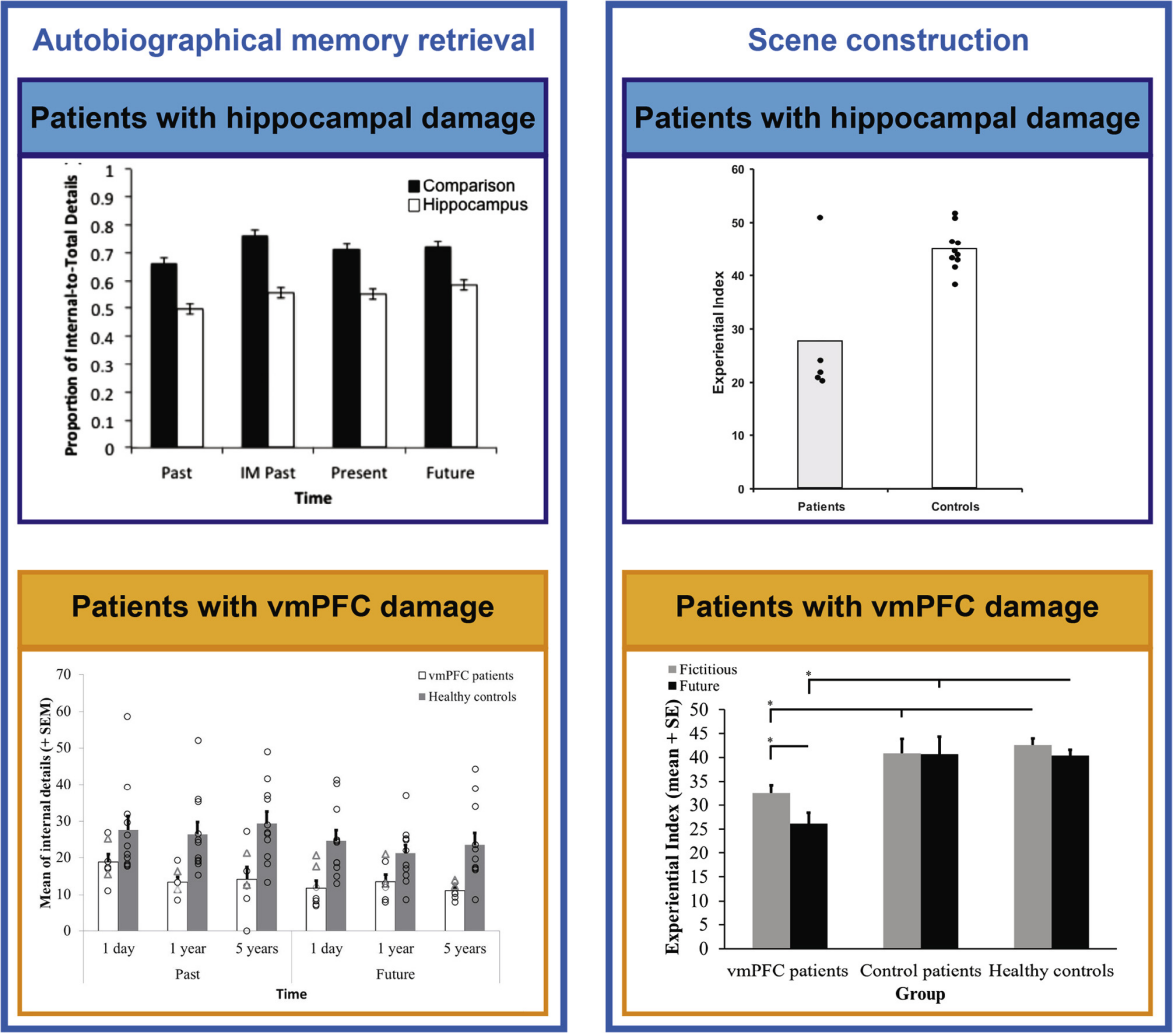
Examples of the poorer performance of patients with hippocampal and vmPFC damage on tasks typically linked with the hippocampus. The left panels show performance of patients with hippocampal (blue) and vmPFC (orange) damage when attempting to retrieve detail-rich autobiographical memories. Both groups show less detailed memory recall than their respective healthy controls. The right panels show performance on a task requiring the mental construction of scenes. Hippocampal (blue) and vmPFC (orange) patients performed poorly on this task relative to their respective healthy controls. The images were adapted with permission from Bertossi et al., 2015, Bertossi et al., 2016, Hassabis et al., 2007 and Kurczek et al. (2015).

Did Phineas Gage (PG) become as amnesic as HM after his accident? In his report from 1868, Harlow notes that two weeks after the accident (at a time when it was very clear that HM was profoundly amnesic), PG recognized his mother and uncle, remembered several people who had visited him, and a number of incidents that had happened since the accident (Harlow, 1869). From these 150-year-old snippets, we can already start to infer a functional difference between patients with vmPFC and hippocampal damage. However, as there were no specific neuropsychological assessments in place at that time (Macmillan, 2000), more fine grained memory problems could have been overlooked. In fact, some studies since then have reported a dense amnesia following vmPFC damage (Della Sala et al., 1993, Kopelman et al., 1999), whereas others reported minimal impairment (Gilboa et al., 2009, Kurczek et al., 2015). Reviewing the literature (see also Fig. 3), which we summarize below, helps to shed some light on this issue.

One hundred years after the case of PG, researchers finally started to examine prefrontal cortex contributions to autobiographical memory. These early studies, albeit involving extensive lesions, showed that patients with PFC damage had significant difficulty recalling autobiographical memories (Della Sala et al., 1993, Kopelman et al., 1999). For example, a common finding at that time was that patients with frontal lesions that included the vmPFC retrieved fewer autobiographical memories than healthy controls. Further, this autobiographical memory retrieval deficit was shown to be strongly correlated with executive functions (i.e., planning, execution of plans) rather than performance on standard memory tests (Della Sala et al., 1993). These findings led to the idea that vmPFC damage might impair retrieval strategies or the organization of autobiographical memory (Moscovitch, 1995, Moscovitch and Melo, 1997), a view that remains prominent (Gilboa, 2010, Gilboa and Marlatte, 2017).

Several lines of research support this interpretation. Patients with PFC lesions, in addition to producing fewer events than controls, displaced their memories along a time-line to a greater degree than patients with MTL damage (Tranel and Jones, 2006). Furthermore, a prospective study on autobiographical memory tried to dissociate patients with PFC (including one patient with selective vmPFC damage) and MTL lesions by sampling sixteen events over an extended period of time and then, in a follow up interview, assessed the number of recalled events and the strategies used for retrieval (Thaiss and Petrides, 2008). Whereas patients with MTL damage recalled generally fewer events and with much less detail than healthy controls, patients with PFC damage recalled slightly fewer events during free recall than healthy controls and did not use spontaneous strategies to order events. However, once they retrieved an event, they were able to produce as much detail as controls.

An important point to bear in mind when trying to interpret disparate findings across studies, is the manner in which autobiographical memory is assessed. Early studies tended to use the Crovitz Test (Crovitz and Schiffman, 1974) which involves 20 cue words and asking participants to retrieve a memory for each one. This is in contrast to later approaches where fewer memories are selected but are analyzed in more depth – such as the autobiographical memory interview (Kopelman, 1994) and the autobiographical interview (Levine et al., 2002). In addition, these later assessment methods provide specific cues when participants have difficulty generating events. So although in-depth analysis has revealed some subtle differences in the quality of autobiographical memories (Rosenbaum et al., 2008, Steinvorth et al., 2005), this may have masked more significant general retrieval or strategic problems that have been hypothesized to accompany vmPFC damage.

An interesting dissociation between intact and impaired autobiographical memory retrieval in vmPFC patients was reported recently (Bertossi et al., 2016, Kurczek et al., 2015, Fig. 3). Kurczek et al. (2015) contrasted patients with bilateral vmPFC and MTL damage by having them first produce six autobiographical memories using a Crovitz-type technique, and then instructed them to select one moment from a memory and describe this in detail. Patients with vmPFC damage could describe these snapshots, or scenes, in as much detail as controls. Individuals with MTL damage, by contrast, were unable to describe single moments from events in vivid detail. In another study, Bertossi et al. (2016) elicited nine autobiographical memories using a Crovitz approach. Having pinpointed a memory, they asked the participants to immediately describe the full event, which could span minutes or hours but less than a day, in as much detail as possible. They found a striking impairment associated with vmPFC damage compared to controls, where patients could not recall in much detail what had happened during the events. This deficit was apparent across both recent and remote autobiographical memories. Therefore, it seems that individuals following hippocampal damage have difficulty conjuring up even one scene in their mind’s eye, while those with vmPFC damage might be impaired in visualizing how extended events unfold.

In summary, as yet it is difficult to come to a firm conclusion about whether autobiographical memory is impaired following vmPFC damage. Accepting that results might be influenced by non-selective lesions and/or possible disconnections, nevertheless, there seems to be a difference between patients with hippocampal and vmPFC damage in their ability to retrieve autobiographical memories. Whereas patients with hippocampal damage recall mostly semantic memories that lack episodic detail, patients with vmPFC damage seem to have a deficit in the generation of autobiographical memories that is particularly evident if entire, extended events (as opposed to single moments/scenes) are probed. Difficulties with schema may have relevance here, which we will consider later. Similarly, problems with autobiographical memory following vmPFC damage may also take the form of confabulation, a phenomenon that we will discuss shortly.

### Future-thinking and scene construction

#### Patients with hippocampal damage

Following on from studies of memory, an interesting extension of this work was the finding that patients with hippocampal damage were also unable to vividly envision their future (Hassabis et al., 2007, Klein and Loftus, 2002, Kurczek et al., 2015; but see Squire et al., 2010 and Dede et al., 2016). It seemed that ‘mental time travel’ (Tulving, 1983, Tulving, 1985, Tulving, 2002) was impaired both backward and forward in these patients (Fig. 3). Since vivid visualizations of scenes feature prominently when recalling the past and imagining the future, it was hypothesized and then confirmed that patients with hippocampal damage could not even imagine fictitious scenes without any requirement for mental time travel (Hassabis et al., 2007; but see Squire et al., 2010). This study indicated that the hippocampus may not support mental time travel per se, but rather the mental construction of the spatially coherent scenes that underpin it (see Ekstrom and Ranganath, 2017 for more on space and time). This finding led to the proposal of the scene construction theory which posits that the hippocampus constructs spatially coherent mental scenes in which details can be bound to be re- or pre-experienced (Clark and Maguire, 2016, Hassabis and Maguire, 2009, Maguire et al., 2016, Maguire and Mullally, 2013, Zeidman and Maguire, 2016; but see Kim et al., 2015, Kim et al., 2011 for an alternative view that the hippocampus exclusively supports memory).

As well as patients with bilateral hippocampal damage being impaired at constructing scenes (Hassabis et al., 2007), other work supports the scene construction theory (Aly et al., 2013, Hannula et al., 2015, Hannula et al., 2006, Hassabis et al., 2007, McCormick et al., 2017, Mullally et al., 2012). For example, boundary extension is a cognitive phenomenon that leads healthy controls, when viewing scenes, to automatically extrapolate beyond the view (Intraub et al., 1992, Intraub and Richardson, 1989). Imagining what lies beyond the boundaries requires the ability to mentally construct scenes, and patients with hippocampal damage and a scene construction deficit showed attenuated boundary extension (Mullally et al., 2012; but see Kim et al., 2015). Furthermore, patients with hippocampal damage have no difficulty detecting differences between faces or objects when they are presented from different viewpoints. However, they are impaired on the same task when scene images are used, because judging scenes from different viewpoints requires the mental construction of scenes (Lee et al., 2005a; but see Kim et al., 2011). We took this further in a recent study, using a new task that dissociated semantic from constructive scene processing. Participants had to detect either semantic violations (e.g. an elephant with butterfly ears) or constructive violations (e.g., an endless staircase) when viewing scenes, where the latter requires the internal representation of the scenes. We found that patients with selective bilateral hippocampal damage successfully detected semantic violations but were impaired at detecting constructive violations (McCormick et al., 2017). Together, these findings show that the ability to construct coherent models of mental scenes is impaired following hippocampal damage (see Clark and Maguire, 2016 for a review).

#### Patients with vmPFC damage

Given the relatively recent linking of the hippocampus with episodic future thinking and scene construction, there are currently only three studies of patients with vmPFC damage assessing their ability to imagine fictitious and future scenes. All three reported deficits in the patients (Bertossi et al., 2015, Bertossi et al., 2016, Bertossi et al., 2017, see Fig. 3). In one study, patients with vmPFC damage were tested using a Crovitz-based autobiographical memory task that was extended to include future events (Bertossi et al., 2016, Crovitz and Schiffman, 1974) and the resulting narratives were scored using a standard autobiographical scoring method (Levine et al., 2002). They found that patients with vmPFC damage were as impaired at imagining future events as they were for past events. In another study, Bertossi et al. (2015) used a scene construction task (Hassabis et al., 2007) in which either fictitious scenes were described (e.g., a bustling market) or future events (e.g., what will you do next weekend?). Again, they found that the vmPFC patients were impaired at imagining both. Lastly, the same authors went on to dissociate the mental construction of future scenarios from describing a picture in plain view or describing a picture they had just seen (Bertossi et al., 2017). They found that patients with vmPFC damage were impaired at providing specific details for all conditions, although controlling for performance in the description conditions did not eliminate vmPFC patients' deficit in the mental construction of future scenarios.

These findings converge with early studies on autobiographical memories suggesting that patients with vmPFC damage might have difficulty generating memories or details (Della Sala et al., 1993, Kopelman et al., 1999). The authors of the three vmPFC studies examining future/fictitious scenes note that the impairment in providing details seemed to be most pronounced during the construction of future scenarios, compared to construction of fictitious scenes and scene descriptions (Bertossi et al., 2015, Bertossi et al., 2016, Bertossi et al., 2017). Whereas the authors interpret this finding as an additional inability to travel mentally in time, it could also be that envisioning past and future scenarios draws more heavily on processes that involve generating coherent visualizations of events that unfold in the mind’s eye. Indeed, in the text examples provided by the authors (Bertossi et al., 2015, Bertossi et al., 2017), descriptions from vmPFC patients mostly consist of momentary snippets, such as a dinner scene, or a market scene. They lack the dynamic unfolding of an event that can be found in the descriptions of healthy controls, such as first we met at the school, we then went home and later prepared something for lunch. This interpretation accords with the finding that, when required to describe a momentary scene from a preselected future event, vmPFC patients provided as much detail as controls, while patients with hippocampal damage provided significantly fewer details (Kurczek et al., 2015).

In summary, on the face of it, it appears that imagining future and fictitious events is dependent on the vmPFC in similar fashion to the hippocampus. However, on closer inspection, the underlying problem might be different. The hippocampus seems to be predominantly involved in constructing individual mental scenes. By contrast, the vmPFC might support the ability to move on from a current scene and progress toward a coherent, mental visualization of an extended event. To date, there are no studies in vmPFC patients on tasks examining boundary extension or the detection of constructive violations in scenes. There is also a need to use fMRI paradigms with patients with hippocampal and vmPFC damage while they are attempting to construct mental scenes and events, in order to examine whether they use remnant hippocampal or vmPFC/PFC tissue when engaged in these complex tasks.

### Navigation and spatial memory

#### Patients with hippocampal damage

Ground-breaking work in the 1970s established that the hippocampus in rodents contains ‘place’ cells and that it plays a key in spatial navigation (O'Keefe and Dostrovsky, 1971, O'Keefe and Nadel, 1978). This discovery has had a profound influence on our understanding of the hippocampus and its function in both animals and humans and stimulated decades of research (Burgess, 2014). From this large literature, we focus here on two exemplar cases of hippocampal-damaged patients that were examined in detail to probe the exact nature of their navigation ability (Maguire et al., 2006, Rosenbaum et al., 2000; see also Teng and Squire, 1999). The first patient, KC (49 years old) suffered severe amnesia resulting from a closed head injury (Rosenbaum et al., 2000, Rosenbaum et al., 2005). His MRI scan showed extensive lesions to both MTLs including the hippocampi, although there were also extensive lesions throughout his brain, including the vmPFC. He performed a number of neuropsychological tasks tapping into various aspects of spatial memory and navigation. Interestingly, he performed comparably to controls on most of the tasks, however, he failed to produce a detailed description of his neighborhood, despite having lived there for almost 40 years.

The other study examined a former London taxi driver, TT (65 years old) who suffered from limbic encephalitis and lost much of his hippocampal function, including the ability to imagine scenes (Hassabis et al., 2007, Maguire et al., 2006). As with KC, TT was able to perform successfully on tasks that included landmark recognition, landmark proximity and distance judgements and pointing to the location of places in London. In another test, using a virtual reality version of central London, TT and control London taxi drivers had to navigate their way from location X to Y. Interestingly, on many of the trials TT got confused and took circuitous routes to the destination, if he arrived at all. It transpired that when TT could use main artery A-routes to get from X to Y he performed well, perhaps relying on semantic memory of these well-worn routes. However, he was unable to reach a goal location if the route demanded that he had to take smaller roads. It seemed that TT could not construct in advance the mental scene of where he had to turn.

#### Patients with vmPFC damage

Surprisingly little is known about the ability of patients with vmPFC damage to navigate in their environment. In fact, there is only one study describing wayfinding in a patient (LG; 56 years old) with vmPFC damage due to a ruptured aneurysm (Ciaramelli, 2008). The author first examined LG’s ability to navigate in his hometown by asking him to find his way from the town center to his work place. Interestingly, LG started on the correct route, but then got distracted and turned a corner to move toward a location where he had worked for 25 years. The author further reports that, when LG arrived at the wrong location, he was able to state where he should have been. Frequent reminders to recall the goal location rescued his navigational difficulties in this ecological test which the author then validated in more formal laboratory tests (Ciaramelli, 2008).

Although not directly assessing navigation, but concordant with the findings in LG, another study (Tranel et al., 2007) contrasted patients with selective vmPFC damage to other PFC lesions on an adapted version of the multiple errands task (Shallice and Burgess, 1991). Participants were asked to enter a shopping mall and complete a list of errands. The authors found that vmPFC patients had more errors and fewer task completions than healthy controls and other patients with PFC lesions outside the vmPFC. Unfortunately, however, the authors do not comment on why the patients did not complete the tasks successfully (i.e., did they get distracted or lost, or did they forget some of the errands). Considering laboratory-based spatial memory tests, patients with vmPFC damage seem to show some specific impairments. For example, on a planning and spatial working-memory task, a computerized version the Tower of London test, patients with PFC damage (including three cases with vmPFC damage) took more trials to sort colored circles than healthy controls (Owen et al., 1990). Furthermore, despite not showing spatial deficits on neuropsychological tasks, individuals with vmPFC damage seemed to use fewer locative words, such as “in”, “around” and “between” (Tranel and Kemmerer, 2004).

In summary, there is a dearth of information about whether and how damage to the vmPFC affects navigational skills. If it does, from the very limited data available, it seems to be a different problem to that described for patients with hippocampal damage. We speculate that patients with hippocampal damage might be impaired at visualizing in advance at which corner they have to turn off the main routes onto smaller routes. By contrast, patients with vmPFC damage may have difficulty initiating mental reminders at critical points in their route planning. Alternatively, their navigation might be adversely affected by perseveration, in the form of an inability to suppress the selection of routes that were relevant in the past, a behavior that is reminiscent of confabulation in the verbal domain. That is, LG might have arrived at a critical junction on his route where he had the choice to either stay on track to follow the new route to his goal or follow a route he traveled for 25 years. Instead of initiating a mental reminder of where he wanted to go, he followed (or got distracted by) familiar locations and old habits.

In reviewing several cognitive functions typically associated with hippocampal integrity, perhaps what is most noteworthy is the substantial gap in our knowledge about how patients with vmPFC damage perform on tasks assessing these functions. Overall, however, we cautiously propose that the vmPFC might be involved in the initiation/generation and/or coordination of dynamic mental imagery in the service of functions such as autobiographical memory, future thinking and navigation. The hippocampus, by contrast, is crucial for the fundamental process of building the single scenes that comprise extended events. We flesh out this idea in more detail later, because we next consider functions typically linked with the vmPFC to examine how hippocampal-damaged patients fare. The same theme is evident, namely, that there are surprisingly few studies testing hippocampal patients on vmPFC-type tasks.

## Functions typically linked with the vmPFC

Twenty years after Phineas Gage’s accident, Harlow published a follow up report, and much of what we know about PG’s cognitive changes is based on this (Harlow, 1869). Harlow noted that “previous to his injury, though untrained in the schools, he [PG] possessed a well-balanced mind, and was looked upon by those who knew him as a shrewd, smart business man, very energetic and persistent in executing all his plans of operation.” After the accident, Harlow reported “he is fitful, irreverent, indulging at times […], manifesting but little deference for his fellows, impatient of restraint or advice when it conflicts with his desires […], devising many plans of future operations, which are no sooner arranged than they are abandoned. […] In this regard his mind was radically changed, so decidedly that his friends and acquaintances said he was ‘no longer Gage’.” PG also developed a “great fondness for pets and souvenirs, especially for children, horses and dogs – only exceeded by his attachment for his tamping iron [which caused the injury], which was his constant companion during the remainder of his life”. This early neuropsychological report indicates that vmPFC damage causes disruptions across cognition including in decision making, emotion, social interactions and personality. We still lack, however, a widely agreed account of precisely what role the vmPFC plays (Abel et al., 2016, Delgado et al., 2016, Szczepanski and Knight, 2014).

### Economic decision making, gambling, and temporal discounting

#### Patients with vmPFC damage

Economic decision making has been closely associated with the vmPFC (Clark et al., 2004, Fellows, 2011, Levy and Glimcher, 2012), with much of the research conducted on gambling (Abel et al., 2016, Clark et al., 2008, O'Doherty et al., 2001, Zald and Andreotti, 2010), often using the Iowa Gambling Test (Bechara et al., 1994). This is a card game where the player has to choose one card at a time from various decks of cards that end up proving either disadvantageous (high risk) or advantageous (low risk). High-risk decks comprise cards that yield high gains but even higher losses, being disadvantageous in the long run, whereas low-risk decks yield small rewards but also small losses. Healthy controls usually learn from past selections, which are accompanied by anticipatory skin conductance responses (SCRs), and they quickly revert to select cards from low-risk decks. In contrast, patients with vmPFC damage do not show anticipatory SCRs, they persist in selecting cards from high-risk decks, and end up gaining less money in the task (Bechara et al., 1996, Waters-Wood et al., 2012). vmPFC patients seem to base their choice of card deck mostly on very recent outcomes, failing to take into account the long-term outcome of their previous choices (Hochman et al., 2010).

Additional evidence of 'myopic' decision making following vmPFC damage comes from studies of delay discounting – the tendency to devalue a reward as the delay until its delivery increases, which may result in preferences for small-immediate over large-later rewards. In the laboratory, delay discounting is assessed by manipulating the time at which different rewards are delivered. For example, a participant may have to choose between £5 now and £15 in a week. The rate at which future rewards are discounted varies across individuals, and correlates with individual differences in real-world behavior, with steep delay discounting generally associated with shortsighted (myopic) behavior. Typically, damage to the vmPFC increases the preference for small-immediate over large-delayed rewards, as revealed by steeper delay discounting of future rewards in vmPFC patients compared with both healthy and brain-damaged controls (Peters and D'Esposito, 2016, Sellitto et al., 2010). Together, these findings provide evidence that the vmPFC supports critical decision making, especially if the task requires integration of information over a longer time period.

#### Patients with hippocampal damage

There have been only a few attempts to formally test value-based decision making in patients with hippocampal damage (Gupta et al., 2009, Gutbrod et al., 2006). Most likely, this is because any differences in decision making that are found between hippocampal patients and healthy controls risk being attributed to patients' dense memory deficit. In fact, on the Iowa Gambling Test, patients with either vmPFC or hippocampal damage had similarly impaired preference learning for the advantageous over the disadvantageous card decks (Gupta et al., 2009, Gutbrod et al., 2006). Importantly, hippocampal patients were unable to develop the normal preference for advantageous decks both when there was a 6-s delay between card selections and when no delay was interposed between card selections, minimizing mnemonic demands. While vmPFC patients tended to prefer high-risk, high-gain decks (Bechara et al., 1994), patients with hippocampal damage did not show a preference for any particular deck (Gupta et al., 2009). Furthermore, whereas patients with vmPFC damage showed barely any changes in SCRs related to the task, hippocampal patients had increased SCRs, displaying significant anticipatory and reinforcement responses (Gutbrod et al., 2006). These findings indicate that whereas patients with hippocampal damage failed to learn the details of the task, which deck was advantageous and which one was not, patients with vmPFC seemed to react impulsively to recent rewards, suggesting that they do not reflect on their overall strategy.

Unlike vmPFC patients, hippocampal-damaged patients have generally shown normal delay discounting rates, indicating they were not abnormally biased toward small-immediate over large-delayed rewards (Kwan et al., 2012, Kwan et al., 2013). Additionally, despite severe problems at imagining future events, patient KC, who suffered from bilateral hippocampal lesions and wider cortical damage, seemed able to think in terms of the future and to consider the distant outcomes of his behavior (Craver et al., 2014). However, whereas healthy controls demonstrated attenuated delay discounting under conditions that required participants first to engage in episodic future thinking (e.g., to imagine spending $42 at a theater in two months) and then engage in delay discounting, hippocampal patients failed to demonstrate this effect, likely because of their difficulty in visualizing appropriate mental scenes (Palombo et al., 2015). As a consequence, in the episodic cueing condition, hippocampal patients had steeper delay discounting than controls, an effect that was also evident when the cues were personally salient (Kwan et al., 2015).

In summary, performance of the two patient groups on gambling tasks appears different. Whereas patients with vmPFC damage make high-risk, high-gain choices, patients with hippocampal damage might fail to learn the hidden rules about which deck is advantageous and which one is not, but they clearly do not gamble. Here, it seems that patients with vmPFC damage react impulsively to high-gain cards, trying to maximize their immediate reward, while failing to initiate the mental processes needed to foreshadow the consequences of their behavior. In agreement with this interpretation, patients with vmPFC lesions explicitly devalue future in favor of immediate rewards. In contrast, patients with hippocampal damage do not show gambling behavior and have preserved delay discounting. Nevertheless, abnormalities emerge if the decision setting encourages the use of mental scenes.

### Moral decision making

#### Patients with vmPFC damage

**Fig. 4:**
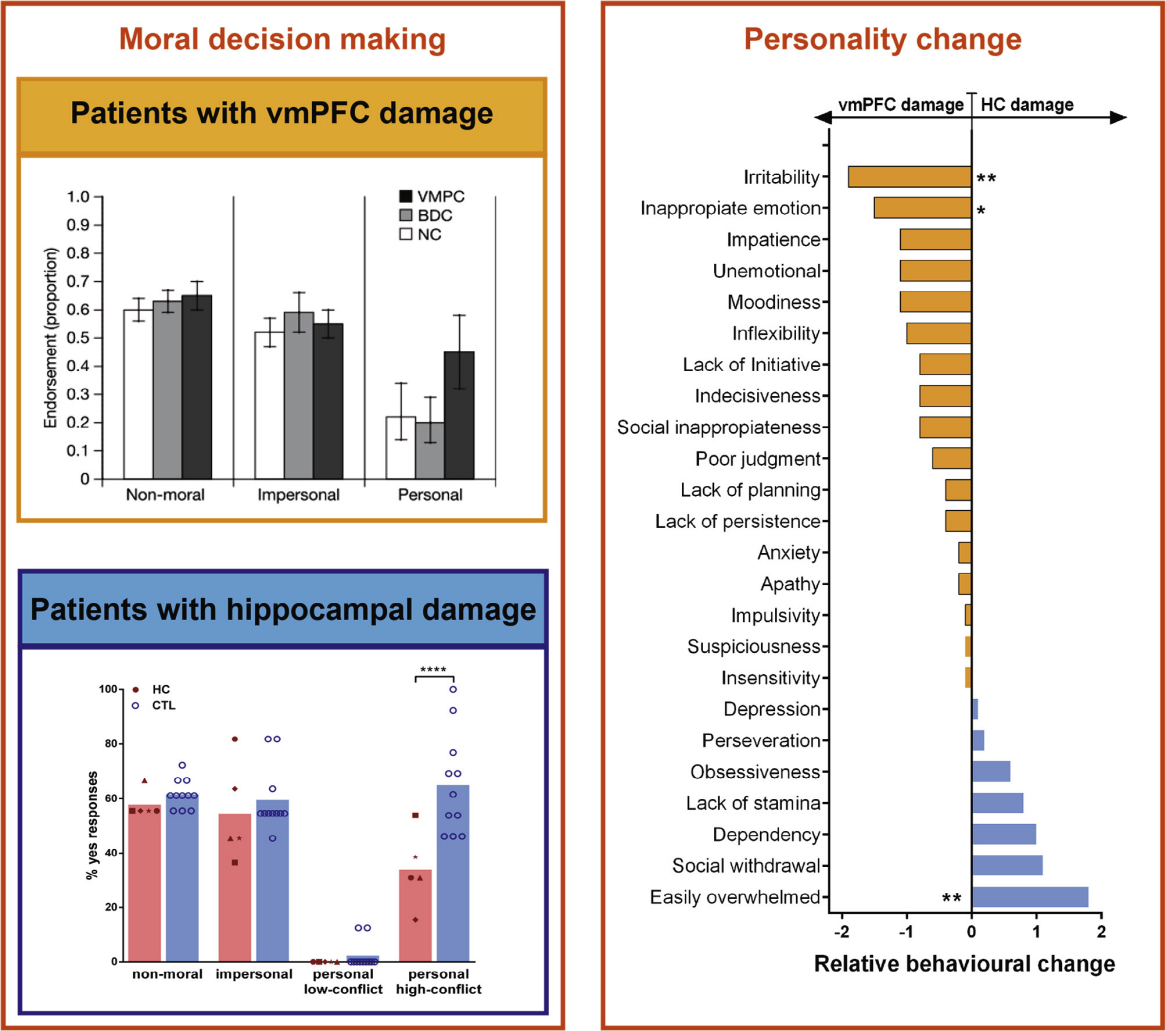
Examples of performance of patients with vmPFC and hippocampal damage on tasks typically linked with the vmPFC. The left panels show the moral decision making of patients with vmPFC (orange) and hippocampal (blue) damage. Whereas vmPFC patients endorsed a utilitarian response more often than controls, patients with hippocampal damage showed the opposite, endorsing more deontological responses. The right panels display the change in personality traits (measured by the Iowa Scales of Personality Change) following vmPFC and hippocampal damage. Whereas patients with vmPFC damage became more irritable and showed inappropriate emotions, patients with hippocampal damage became more socially withdrawn and easily overwhelmed. The images were adapted with permissions from Koenigs et al. (2007) and McCormick et al. (2016). BDC = brain-damaged controls; HC = hippocampal-damaged patients; CTL = control subjects.

Another classic decision making task involves moral dilemmas where the participant has to decide whether to endorse a morally inappropriate action, such as defrauding the tax system or not. Patients with vmPFC damage typically perform normally on these tasks except if the moral dilemma involves causing serious bodily harm to a human being through one’s own agency (i.e., personal moral dilemmas, Fig. 4; Ciaramelli et al., 2007, Ciaramelli et al., 2012, Ciaramelli et al., 2013b, Koenigs et al., 2007). In these extreme dilemmas, where often the decision to be taken concerns whether or not to take somebody’s life with one’s own hands in order to save multiple people, patients with vmPFC choose the utilitarian option more often than controls, are faster at making their decisions and show attenuated SCRs (Ciaramelli et al., 2007, Koenigs et al., 2007, Moretto et al., 2010, Thomas et al., 2011). Another line of research has shown that vmPFC patients fail to consider the intention behind actions, and tend to base their moral judgments predominantly on the outcome of actions, therefore exhibiting an abnormal appraisal of cases involving either attempted or accidental harm (Ciaramelli et al., 2012, Young et al., 2010). Thus, the moral decision making literature overall suggests that patients with vmPFC damage cannot foresee, and emotionally respond to, the future impact of their actions (Levens et al., 2014, Thomas et al., 2011, Young et al., 2010), nor can they consider sources of information removed from their current (perceptual) experiences, such as the intentions of others.

#### Patients with hippocampal damage

Despite the numerous scientific studies conducted on HM, his value-based decision making was not examined thoroughly. However, Corkin describes in a review about HM (Corkin, 2002) that he had “beliefs, desires and values that are always present. […] He is altruistic: when I asked him to tell me about Dr. Scoville he said: ‘He did medical research on people – all kinds of people. What he learned about me helped others too, I’m glad about that.’ ”. Corkin further explains that “his social behavior is appropriate and courteous. […] He has high moral standards with respect to right and wrong in his personal conduct”. From these anecdotes we can infer that there may be differences in value-based decision making between vmPFC and hippocampal patients.

Moral decision making has recently been formally examined in patients with hippocampal damage (Craver et al., 2016, Croft et al., 2010, McCormick et al., 2016; Fig. 4). Interestingly, here also patients with vmPFC or hippocampal damage differ. For example, whereas patients with vmPFC damage hardly changed their opinion about somebody’s character after learning that somebody had done something morally wrong, patients with hippocampal damage changed their opinion dramatically, even to a greater extent than healthy controls (Croft et al., 2010). In a classic moral dilemmas task, we recently found that hippocampal patients approved of the utilitarian options significantly less often than control participants, favoring instead deontological responses – rejecting actions that harm even one person (McCormick et al., 2016; but see Craver et al., 2016). Skin conductance data showed increased emotional arousal in the hippocampal-damaged patients and they stated that their moral decisions were based on emotional instinct. By contrast, control participants made moral decisions based on the integration of an adverse emotional response to harming others, visualization of the consequences of one’s action and the rational re-evaluation of future benefits.

In summary, very little research has been conducted on moral decision making in patients following hippocampal damage. Based on the limited available evidence, hippocampal patients seem to have the opposite approach to moral decision making compared to vmPFC-lesioned patients – the latter are consistently reported to show an overly utilitarian pattern of responding that is devoid of emotion, whereas the former are overly deontological, and overwhelmed by a strong emotional response to violations that are not tempered by visualization of future benefits. It is worth noting that visualization of the moral scenarios is also likely to be reduced in vmPFC patients, given the hypothesized difficulty they have initiating/generating/coordinating the mental imagery of extended events that we outlined earlier. Coupled with their impaired ability to detect emotional responses, this may leave them solely reacting to current (perceptual) external inputs, rendering life and death decisions based merely on the highest number of people that could be saved in that moment.

### Emotion regulation

#### Patients with vmPFC damage

The decision making considered above is inherently linked with the idea that the vmPFC plays a critical role in emotion regulation (Anderson et al., 2006, Bechara, 2004, Beer et al., 2006, Berlin et al., 2004, Drevets et al., 2008, Jonker et al., 2015, Krueger et al., 2009). For example, vmPFC patients are poorer at recognizing emotional faces (Heberlein et al., 2008, Tsuchida and Fellows, 2012) and look less often at the emotionally salient information in faces (Wolf et al., 2014). They also show reduced skin conductance responses to emotional stimuli, such as pictures of emotional faces (Koenigs et al., 2007). Moreover, the commonly used Iowa Scales of Personality Change (ISPC) show that patients with vmPFC damage demonstrate blunted emotional expressiveness (Barrash et al., 2011, Barrash et al., 2000, Fig. 4). Interestingly, on the flip side, vmPFC patients tend to react more passionately (mostly with anger), about task outcomes that conflict with their desires (Koenigs and Tranel, 2007, Krajbich et al., 2009, Shamay-Tsoory et al., 2012, Trebuchon et al., 2013), and have strong stereotypical opinions (Gozzi et al., 2009). It has also been shown that vmPFC patients have a greater disposition to negative mood induction, and more aggressive, impulsive and inappropriate outbursts of behavior (Gillihan et al., 2011). These sudden outbursts of negative emotional behavior are interesting, since lesions to the vmPFC are sometimes described as protective against depressive disorders (Koenigs et al., 2008a, Koenigs et al., 2008b). Although speculative, linking with our suggestions above, this could be because vmPFC patients react to their environment in a direct and impulsive manner with little generation of inner mental reflections, including mental imagery.

#### Patients with hippocampal damage

Little is known about changes in emotional regulation following hippocampal damage (Bach et al., 2014, Beadle et al., 2013). Whereas general autonomic responses seem to be intact in hippocampal patients (Bechara et al., 1995, Gutbrod et al., 2006), emotion induction seems to be elevated (Feinstein et al., 2010, McCormick et al., 2016). That is, after watching sad or happy movie clips, patients with hippocampal damage felt emotional long after they had lost the explicit memory of these movies (Feinstein et al., 2010). Furthermore, in a recent study conducted during moral decision making, we found heightened emotional responses as measured by galvanic skin conductance and debriefing strategies (McCormick et al., 2016). We further showed that patients themselves and their close relatives rated the patients as more emotional and socially anxious following their illness (Fig. 4).

In summary, despite the lack of evidence for changes in emotional regulation following hippocampal damage, it is interesting to note that hippocampal patients differ from vmPFC patients in the quality of their emotions. That is, whereas vmPFC patients are described as impulsive, aggressive and, surprisingly, show little evidence of depression (despite the typically large negative impact of the brain damage on their lives), patients with hippocampal damage seem to score higher on anxiety and depression measures (McCormick et al., 2016). It could be argued that impulsivity and aggression are more connected with overt behavior and involve less introspection, whereas anxiety, depression and sadness are more covert behaviors that may depend more upon reflective mental activity. In agreement with this idea, it has been noted that whereas vmPFC patients typically lack insight into their mental state (Barrash et al., 2000, Del Cul et al., 2009), hippocampal patients generally have insight into their memory problems (Corkin, 2002).

### Social interactions and theory of mind

#### Patients with vmPFC damage

The vmPFC has also been implicated in social cognition, where it is held to support the evaluation and representation of interpersonal qualities, and the ability to infer what is in the minds of other people, also known as theory of mind (Beer et al., 2003, Delgado et al., 2016). The advocates of this view argue that much of the behavioral change following damage to the vmPFC, such as utilitarian decision making, impulsive and aggressive behaviors, occur in social situations. In support of this account, patients with vmPFC damage tend to show inappropriate verbal behavior toward strangers (Rolls et al., 1994), or disclose inappropriate personal information in conversations with strangers without the embarrassment typically associated with such inappropriate social behavior (Beer et al., 2006). Moreover, patients with vmPFC damage endorse more often than healthy and brain-damaged controls behaviors that normally elicit interpersonal disgust (Ciaramelli et al., 2013b), and have abnormal (closer) interpersonal distance preferences (Perry et al., 2016). At the same time, vmPFC patients tend not to hold any close relationships or work in regular employment (Saver and Damasio, 1991), showing a problem in regulating social distance and conduct. Damage to the vmPFC also leads to a significant reduction in the ability to generate effective options to solve real-world scenarios, especially those that are social in nature (Peters et al., 2017). Notably, patients with vmPFC damage seem to have retained semantic knowledge of social rules. For example, in the study by Beer et al. (2006), vmPFC patients felt normal embarrassment when they viewed their videotaped socially inappropriate behavior, suggesting they are aware of social norms, but lack self-insight and online mental reflection about what is appropriate in social situations.

The ability to simulate what other people think or feel, known as theory of mind (ToM), mentalizing, or cognitive empathy, has been related to processing in the medial frontal lobes in humans (Gallagher and Frith, 2003). It has been reported that theory of mind is impaired in patients with vmPFC lesions (Eslinger, 1998, Shamay-Tsoory et al., 2005). For example, vmPFC patients fail on tasks which evaluate one's ability to understand what someone else thinks about what someone else thinks (Shamay-Tsoory et al., 2003). They also have problems detecting faux pas, i.e., situations in which a character says something without considering whether or not the listener might want to hear it, which requires inferring (simulating) other's mental states (Stone et al., 1998). Interestingly, vmPFC patients may show intact emotion recognition and affective empathy which do not require the mental visualization of alternative perspectives and instead rely on immediate emotional contagion and resonance mechanisms (Zaki and Ochsner, 2012).

#### Patients with hippocampal damage

Naturally, social relationships can change if one consistently cannot remember having met a person and opportunities to move around independently in the world are curtailed. It is therefore logical that a patient’s social network reduces following hippocampal damage (Rubin et al., 2014). Above and beyond this fact, however, there is a dearth of information about social cognition following hippocampal damage. Because of their striking memory problem, many changes in the social domain following hippocampal damage might have been attributed solely to their memory problem and therefore not thoroughly examined. Moreover, outward emotional changes, such as the aggressive or grossly inappropriate behavior associated with vmPFC damage, are not evident in hippocampal patients, and so perhaps did not invite further study. Hence, research has just begun to investigate the interpersonal relationships of patients with hippocampal damage with indications that the social interactions in these patients are altered post-illness.

In a single case report, Warren et al. (2012) described the life of a woman with hippocampal amnesia who managed, with much support from her husband and parents, to raise her two children and retain a few close social bonds. This, and a few other cases, seems to suggest that there can be positive life outcomes after bilateral hippocampal damage (Corkin, 2002, Duff et al., 2008). However, these reports did not mention that living with a severely memory-impaired person can be a tremendous challenge for close family members. As described above and also mentioned by the case reports, patients with hippocampal damage seem to become extremely careful in social situations (McCormick et al., 2016, Warren et al., 2012) and sensitive to emotional stimuli (Feinstein et al., 2010, Gutbrod et al., 2006). This aligns with their higher depression and anxiety scores (Warren et al., 2012) which, interestingly, is a common comorbidity with patients who have medial temporal lobe epilepsy (Bell et al., 2011). Social interaction also requires the ability to imagine and reflect upon experiences with other people, a function that is reduced in patients with hippocampal damage. In agreement with this, Sheldon et al. (2015) reported that patients with MTL damage came up with fewer solutions to open-ended problems than controls.

Hippocampal patients perform comparably to controls if they are asked to infer the mental states of others in theory of mind tests, including false belief tasks, faux pas detection, and emotion recognition (Rosenbaum et al., 2007). It is possible that hippocampal patients are not impaired on these tasks because these may be solved using semantic knowledge or rationalizing about how the 'average' person may feel in a particular situation, whereas episodic memory may be necessary to tailor this ability to specific social targets (Ciaramelli et al., 2013a). Intriguingly, a patient with developmental amnesia was able to infer the experiences of unfamiliar others, but was impaired at inferring those of familiar others, which may depend more heavily on access to episodic memory (Rabin et al., 2012).

In summary, there are clear differences between patients with vmPFC and hippocampal damage in their social interactions and ability to infer the mental states of others. In line with our suggestions above, vmPFC patients seem to react generally to their current (perceptual) environment in a rapid and impulsive manner devoid of mental reflection, whereas patients with hippocampal damage have more insight into their deficits, and are understandably socially nervous. This interpretation is in agreement with theory of mind studies, which consistently demonstrate a lack of mental insight in vmPFC patients, while this is generally preserved in hippocampal patients, as long as the task does not require the visualization of mental scenes.

### Counterfactual thinking

#### Patients with vmPFC lesions

Counterfactual thoughts are mental simulations of what might have been if another behavior had been executed. They are pervasive in everyday life, help people learn from experience, modulate their emotional state, and contribute to decision making and social functioning. Consistent with the long-known impaired mental insight of patients with prefrontal lesions, evidence suggests that the frontal lobes may be involved in the generation and use of counterfactual thinking. For example, Knight and Grabowecky (1995) described a man with a PFC lesion who showed a complete absence of spontaneous counterfactual expressions, in spite of the experience of emotional stressors. It has also been reported that frontal patients generated fewer thoughts of regret and difficulty in learning from their own experiences (Camille et al., 2004). PFC patients’ ability to generate counterfactual thinking spontaneously (e.g., recalling a negative event in the past and reporting what they were thinking about it right now) has been found to be reduced compared to controls. In contrast, there were no differences between PFC patients and controls when counterfactual thinking was guided by specific questions (Gomez Beldarrain et al., 2005). While the PFC has been suggested as a critical brain region for counterfactual thinking, surprisingly, there is no study directly examining this form of thought in patients with selective vmPFC damage.

#### Patients with hippocampal lesions

Given that episodic memory and imagining the future are impaired in patients with hippocampal damage and amnesia, Mullally and Maguire (2014) tested whether counterfactual thinking depends upon the integrity of the hippocampus. In two non-episodic counterfactual thinking tasks (i.e., tasks not based on memory for one's personal past), they found that patients with bilateral hippocampal damage and amnesia performed comparably to matched controls. They could deconstruct reality, add in and recombine elements, and change relations between temporal sequences of events, enabling them to determine plausible alternatives of complex episodes. A difference between the patients and control participants was only evident in the patients' subtle avoidance of counterfactual simulations that required the construction of an internal spatial representation.

In summary, these findings suggest that PFC (and possibly vmPFC, although clear evidence is lacking) patients have problems with generating internal representations of alternative extended events, potentially echoing their difficulties in autobiographical memory and possibly future-thinking. In contrast, mental simulation in the form of counterfactual thinking does not seem to depend upon the hippocampus, unless there is the added requirement for construction of a coherent spatial scene within which to play out mental scenarios.

### Mind-wandering

A recurrent theme of this review is, on one hand, the inability of patients with vmPFC damage to initiate internal reflections, including mental visualizations of extended events, and on the other hand hippocampal patients seem able to initiate mental events but they are devoid of visual representations of scenes. With this in mind, the ability to decouple from the current environment and let one’s mind wander is of significant interest. That is, mind-wandering only occurs upon the initiation of mental events (Callard et al., 2013, Smallwood and Schooler, 2015), and prominently involves mental imagery of scenes (Andrews-Hanna et al., 2013, Smallwood et al., 2016), such as autobiographical reminiscences, future-thinking and atemporal scene and event simulations (Andrews-Hanna et al., 2014, Baird et al., 2012, Baird et al., 2011). Unfortunately, as yet we know little about the effects of vmPFC or hippocampal damage on mind-wandering. Nevertheless, preliminary evidence from two studies suggests that, once again, both brain structures play important yet distinct roles in this cognitive function.

#### Patients with vmPFC damage

One of the first clinical observations of patients with prefrontal lesions was of “Spontanstummheit” (Kleist et al., 1940), which denotes a lack of spontaneous mental activity. In a recent study, Bertossi and Ciaramelli (2016) examined mind-wandering in patients with vmPFC damage. A series of computer tasks varying in difficulty and conduciveness to mind-wandering was used (e.g., paying attention to digits on the computer screen and judging whether the current or previous digit was even/odd). Across tasks, patients with vmPFC damage were asked five times whether their thoughts had been on or off task, and about the contents of their thoughts. They found that patients with vmPFC damage showed a reduced frequency of mind-wandering and, on the occasions when mind-wandering had occurred, there was a reduced focus on future-oriented thoughts and an increased focus on present-related thoughts. These findings are the first formal indication that damage to the vmPFC alters the frequency and temporal focus of mind-wandering. The spouse of a vmPFC patient mentioned that her husband from time to time made remarks about things in the (external) environment that grabbed his attention, but he never mentioned any thoughts that were internally motivated, such as a thought or emotion that had occurred to him spontaneously. In line with the recurrent theme of this review, that the vmPFC might initiate mental imagery, the authors interpreted their findings as indicating a potential difficulty for vmPFC patients in directing attention inward, possibly due to deficits in generating mental scenarios.

#### Patients with hippocampal damage

One study examined mind-wandering in patients with selective bilateral hippocampal damage in an extended experimental setting (McCormick et al., preprint). The authors shadowed patients with selective bilateral hippocampal damage for two days and asked them on twenty different occasions what they had been thinking about just before the experimenter had asked them. They found that patients with hippocampal damage reported as much mind-wandering as controls, however, the form and content was markedly different. Whereas controls thought flexibly about the past, present or future, using vivid, visual scenes, the thoughts of the patients were constrained to the present, mainly containing verbal descriptions about themselves or the world around them.

In summary, there is a clear need for more studies investigating the similarities and differences in mind-wandering in patients with hippocampal and vmPFC damage. Furthermore, while there are numerous differences in the task designs of these two mind-wandering studies that may have influenced their findings, the results are in line with the emerging theme. That is, patients with vmPFC damage seemed impaired at decoupling from the external environment and initiating or generating vivid mental scenarios. In contrast, patients with hippocampal damage mind-wandered as much as controls, but seemed to have difficulty visualizing the coherent scenes that constitute the backbone of mind-wandering experiences in healthy controls.

### Memory monitoring, and its failure in confabulation

#### Patients with vmPFC damage

From the earliest neuropsychological studies of patients with frontal lobe lesions, a curious phenomenon was reported, that of confabulation (Kleist et al., 1940). Confabulation, sometimes referred to as “honest lying” (Moscovitch, 1989), is the unintentional production of false memories. Interestingly, Harlow mentioned in his 1868 report that Phineas Gage’s mother told him that he got accustomed to entertaining his young nephews and nieces with the most extraordinary recitals of his escapades without any foundation in reality, indicating that PG might also have confabulated. Spontaneously, or upon questioning, patients with frontal lesions sometimes narrate, typically with conviction, events that have never happened, or are not of current relevance (Gilboa et al., 2006a). Some confabulating patients even act upon their confabulatory beliefs (e.g., leaving by train to reach a job they used to do when they were young).

Systematic neuropsychological work has linked confabulation to the vmPFC, especially the most posterior part of vmPFC, including the basal forebrain (Gilboa, 2010, Gilboa et al., 2006a, Moscovitch and Melo, 1997). The symptom of confabulation is important to current theories about the role of the vmPFC in memory processes. It led to the idea that the vmPFC plays a critical role in the strategic retrieval of memories (Gilboa et al., 2006a, Gilboa and Marlatte, 2017, Moscovitch and Melo, 1997). In addition, the high confidence with which patients confabulate initiated a debate about the role of the vmPFC in mental insight (Gilboa, 2010, Hebscher and Gilboa, 2016). From this perspective, confabulation is seen as an aberrant process where memories are incorrectly retrieved and linked with current demands. In this regard, schemas began to emerge as important constructs. These are adaptive knowledge structures that comprise associative information that is acquired over multiple episodes (Ghosh and Gilboa, 2013). In line with such ideas, patients with vmPFC damage are impaired in deciding between schema-relevant and irrelevant information (Ghosh et al., 2014). Furthermore, schemas could provide the backbone for the extended mental events that seem missing in the autobiographical memory descriptions of patients with vmPFC damage (Bertossi et al., 2016, Kurczek et al., 2015). Moreover, on word lists such as those used in the Deese–Roediger–McDermott paradigm that contain highly schematic information (thematically related words; e.g., hill, valley and range), healthy controls usually produce false-positive responses for highly schema-congruent words (e.g., mountain; Roediger and McDermott, 1995). Patients with vmPFC damage make significantly fewer errors on this task (Ciaramelli et al., 2006, Warren et al., 2014). Similarly, in autobiographical recognition memory tasks, vmPFC patients with confabulation endorse even highly implausible lures related to autobiographical events, which are blatantly inconsistent with self-schema (Gilboa et al., 2006a).

#### Patients with hippocampal damage

To the best of our knowledge, there is no equivalent of confabulation in patients with hippocampal damage. While these patients might get individual facts wrong, their narratives are in themselves consistent. Surprisingly little is known about schemas in patients with hippocampal damage. They are able to rehearse commonly known fairy tales, such as red riding hood, and other autobiographical semantic stories (Gilboa et al., 2006b, Rosenbaum et al., 2009, Verfaellie et al., 2014), yet patients with MTL damage seem to perform in a similar manner to vmPFC patients on the Deese–Roediger–McDermott paradigm (Chiu et al., 2010, Melo et al., 1999, Schacter et al., 1997, Verfaellie et al., 2002), although this has not been tested in patients with selective bilateral hippocampal damage. There is evidence, however, that in recognition memory tasks, amnesic patients with medial temporal lesions, unlike vmPFC patients, do not have problems at distinguishing currently relevant from previously encountered but currently irrelevant information, confirming preserved memory monitoring (Schnider and Ptak, 1999).

In summary, there is still much to learn about confabulation in vmPFC patients, and it is clear that schema research needs to be conducted with hippocampal patients. In addition, while schemas may play a role in confabulation in vmPFC patients, findings could also be interpreted as scene descriptions that lack the coherent dynamic embodiment of a mental event. That is, little is known about whether confabulations are visualized or devoid of mental imagery, and whether they contain dynamic information or are scene snapshots. Furthermore, based on one of the author’s clinical observations (EC), and as also alluded to by Gilboa et al. (2009), confabulation can decrease over time in some vmPFC patients. However, it is unclear why it decreases and whether or why they stop acting upon confabulatory beliefs. Are patients still internally confabulating but have learned to mistrust, and so not articulate and act upon, their thoughts? Could this be inhibiting their performance on autobiographical memory and scene construction tasks?

By reviewing several cognitive functions typically associated with vmPFC integrity, it becomes clear that much more work is needed in order to come to firm conclusions about how patients with hippocampal damage fare on tasks assessing these functions. Overall, however, disparities between the two patients groups emerge much more dramatically on classic vmPFC tasks compared to cognitive functions typically associated with the hippocampus. So far, the preliminary picture is that patients with vmPFC damage generally lack the ability to initiate or generate mental reflections which are needed to make informed decisions and react to current emotions in a reasonable fashion that incorporate future consequences. Instead, this impairment seems to render these patients reactive to current (perceptual) inputs, so that their behavior appears impulsive and aimed at maximizing their immediate reward. In contrast, patients with hippocampal damage seem to be able to mentally reflect upon themselves and their surroundings, however, these mental events are devoid of visualizations. It is further interesting to note that, in some circumstances, hippocampal patients seem to perform in an opposite fashion to patients with vmPFC damage, such as during moral decision making or social interactions. These results indicate that a vmPFC in the context of afunctional hippocampi might overreact, especially in emotional situations where mental visualizations might be crucial to foreshadow future consequences.

## Conclusion and a proposal

In the first half of this review we focussed on patients with vmPFC damage performing tasks that are typically impaired in patients with hippocampal lesions. In the second half, we focussed on hippocampal-damaged patients performing tasks that are usually impaired in vmPFC patients. A general summary of the impact of hippocampal or vmPFC lesions is shown in Table 1, with the caveat that some findings may have been influenced by non-selective lesions and possible structural or functional disconnections.

**Table 1:** Summary of cognitive changes following hippocampal and vmPFC damage

The blue section illustrates functions commonly associated with the hippocampus and the orange section functions commonly associated with the vmPFC. The arrows indicate that the groups behave differently compared to healthy controls. Specifically, “↓” refers to a functional decrease for that group, for example patients with hippocampal damage typically have problems recalling autobiographical memories. In reference to this phenotype, the other group can be classified as “↓” meaning the impairment in general follows the same direction, i.e., vmPFC damage also causes impairments on autobiographical memory retrieval. The additional “≠” indicates that the underlying reasons for the deficits seem different. On the other hand, “↑” indicates a functional increase for that group, for example patients with vmPFC damage discount more future rewards in preference for immediate rewards compared to controls. Again, the additional “≠” indicates that there are differences between the patients groups, for example patients with hippocampal damage show normal delayed discounting, except if visualizations are required. “Preserved” indicates that this function is similar to that of healthy controls. ‘?’ indicates that the evidence is not completely clear, given a lack of lesion specificity.

Three points in particular are notable. First, there are large gaps in our knowledge about how hippocampal and vmPFC patients perform on tasks that are typically associated with the other group, and evidence is especially lacking in patients with selective lesions to the vmPFC. This is surprising, given the many decades spent examining patients with lesions in the hippocampus and vmPFC, and the strong connections between these two brain areas. While there have been recent advances in examining functions typically associated with the vmPFC in patients with hippocampal damage, more research needs to be conducted on testing vmPFC patients on tasks typically associated with hippocampal function.

Second, while patients with either hippocampal or vmPFC damage seem to perform similarly on so-called hippocampal tasks, the two patient types diverge significantly on classic vmPFC tasks. For example, whereas lesions to the hippocampus and vmPFC reduce the ability to retrieve vivid autobiographical memories (Bertossi et al., 2016), and construct mentally coherent events (Bertossi et al., 2015, Bertossi et al., 2017), lesions to the hippocampus and vmPFC seem to have very different, even opposite, effects on personality, emotion regulation, economic and moral decision making and social interactions. Patients with vmPFC lesions tend to become utilitarian, impulsive and socially inappropriate (Ciaramelli et al., 2007, Koenigs et al., 2007), whereas hippocampal patients have high moral standards, become socially nervous (Corkin, 2002, Croft et al., 2010, McCormick et al., 2016), and have veridical, though impoverished, memories.

Third, although the performance of the two patient types appears analogous on hippocampal tasks, on closer inspection, there are disparities between hippocampal and vmPFC patients. We now consider these differences further as part of a tentative proposal about what these two areas might be doing and how they interact.

We suggest that the hippocampus and vmPFC might align in a hierarchical network, in which the hippocampus plays a subordinate role. While the consequences of hippocampal damage are dramatic and affect almost every aspect of one’s life (Duff et al., 2008, Warren et al., 2012), the deficit, if the lesions are selective to the hippocampi, seems to primarily reside in an inability to mentally construct spatially coherent scenes (Clark and Maguire, 2016, Hassabis et al., 2007, Maguire and Mullally, 2013). As several lines of research have shown, hippocampal scene-based information is crucial to much of our cognition, including autobiographical past and future-thinking (Kurczek et al., 2015), scene perception (McCormick et al., 2017), mind-wandering (McCormick et al., preprint) and decision making (McCormick et al., 2016). Moreover, recent neuroimaging work in healthy controls has specifically implicated the anterior hippocampus in scene construction (Zeidman et al., 2015a, Zeidman et al., 2015b, Zeidman and Maguire, 2016), which makes sense given this part of the hippocampus is known to be particularly well connected with the vmPFC (Adnan et al., 2016, Catani et al., 2013, Catani et al., 2012). Crucially, we have recently shown that patients with hippocampal damage can detect semantic violations in scenes but not constructive violations (McCormick et al., 2017). This finding indicates that processing a collection of objects within a space or understanding the semantic content of scenes does not require hippocampal input. What seems to drive hippocampal involvement is the construction of a spatially coherent mental scene into which details can be bound in order to be re- or pre-experienced.

We propose that the vmPFC acts as a supervisor that initiates endogenous processes and in particular scene construction. While data supporting this idea are limited at present, cognitive changes following vmPFC damage support this idea. The lack of mind-wandering episodes following vmPFC is especially interesting because it indicates an issue with initializing mental decoupling (Bertossi and Ciaramelli, 2016). Furthermore, common personality changes, such as impulsive, aggressive and socially inappropriate outbursts converge on the idea that individuals with vmPFC damage can react to external stimuli, but might be unable to initiate appropriate endogenous reflection, which in healthy individuals often involves scene imagery. The autobiographical memory findings are also concordant with this view - vmPFC patients fail to generate as many autobiographical event memories as healthy controls during recall tasks (Bertossi et al., 2016, Della Sala et al., 1993, Kopelman et al., 1999). Especially notable in this regard is the preserved ability of vmPFC patients to describe in detail single snapshot scenes from these memories (Kurczek et al., 2015).

We posit that the vmPFC works with the hippocampus by initiating the scene construction process, perhaps coordinating the curation of relevant elements from neocortical areas, which are then funneled into the hippocampus to build a scene. The vmPFC then engages in iterative re-initiation via feedback loops with neocortex and hippocampus to facilitate the flow of multiple scenes that comprise the coherent unfolding of an extended mental event. Indeed, in healthy individuals, the magnetoencephalography (MEG) phase coherence between vmPFC and hippocampus is significantly stronger during dynamic scene exploration than static scene exploration (Kaplan et al., 2017). A predicted consequence of this interaction between the two brain areas is that remote autobiographical memories should involve the vmPFC to a greater extent than recent memories, which has been confirmed in fMRI studies of healthy controls (Bonnici et al., 2012, Bonnici and Maguire, 2017). We believe this is because remote memories have already been consolidated to the neocortex, and so their retrieval requires more initializing, coordinating and iterating on the part of the vmPFC to ensure appropriate re-construction of the events.

The vmPFC receives direct visual input from visual-perceptual areas via the inferior fronto-occipital fasciculus (Catani et al., 2012). These strong anatomical connections place the vmPFC in an ideal context to initiate a scene construction process. In fact, whereas electrophysiological power changes can be seen almost immediately after stimulus onset in the vmPFC and remain there until the end of the stimulus (Sederberg et al., 2007), power changes in the hippocampus occur much later, around 500–2000 ms after stimulus onset (Sederberg et al., 2003, Sederberg et al., 2007). These ideas align with recent electrophysiological findings in healthy controls and vmPFC patients of frontally mediated rapid memory processes (Gilboa and Moscovitch, 2017), and an MEG study using directional connectivity analyses showing that the vmPFC influenced match–mismatch responses in the hippocampus (Garrido et al., 2015). Therefore, we propose that the vmPFC drives hippocampal scene construction processes during autobiographical memory retrieval, future-thinking and navigation.

Confabulation might, from this perspective, be viewed as remnants of hippocampal scene construction processes that gain access to consciousness without a supervisor who is equipped to deal appropriately with scenes and coordinate the appropriate transitions between contiguous scenes. In contrast, patients with hippocampal lesions have an intact supervisor that can initiate endogenous reflection. Whereas in healthy controls the vmPFC integrates mental scenes into vivid detail-rich dynamic events, the scenes themselves are missing in patients with hippocampal damage leading to an over-representation of abstract mental reflection. Indeed, mind-wandering episodes of hippocampal patients have been shown to consist mainly of abstract, self-reflective verbal thoughts (McCormick et al., preprint).

## Future directions

Our proposal is, of course, tentative. To be tested properly, the significant gaps in knowledge that we highlighted throughout the review need to be filled. Ideally this would involve examining patients with more selective, well-characterized lesions. While great strides have been made in using high resolution MRI to characterize hippocampal lesions and connectivity (Addis et al., 2007a, Maguire et al., 2001, Miller et al., 2017, Mullally et al., 2012, McCormick et al., 2014, McCormick et al., 2013, McCormick et al., 2016, McCormick et al., 2017, Rabin et al., 2016), this is much more challenging for the vmPFC. Patients with vmPFC damage often have contra-indications for MRI. It may be that very rare cases with acute, MRI-compatible pathology, where lesion selectivity and the intactness of salient white matter tracts can be verified, may ultimately be the most informative.

It is likely that many cognitive domains remain relatively underexplored in patients with hippocampal damage because of their severe amnesia. However, these patients offer a unique opportunity to study the rest of the brain and how it copes with hippocampal damage. For instance, what does the vmPFC do under circumstances in which hippocampal input is absent? Might it result in a hippocampal patient being overly sensitive in the context of moral judgments? Moreover, do patients with hippocampal damage respect interpersonal space? Perhaps they allow an abnormally large amount of space between themselves and others. Do patients with hippocampal damage have an inconsistent hierarchy of subjective values or do they, in fact, have a very rigid system of values? Also, would patients with hippocampal damage shy away from taking any risks on gambling tasks if they were explicitly told which card deck holds high- and low-risk cards?

In contrast, patients with vmPFC damage offer a unique opportunity to study the brain with a functioning hippocampus but without a supervisor. An obvious question in this instance is whether vmPFC patients can construct single scenes but are impaired if they have to initiate more elaborate endogenous processing? One way to address would be to use our task where participants have to detect semantic and constructive violations in scenes (McCormick et al., 2017). It may be that vmPFC patients, unlike those with hippocampal damage, are able to perform normally, since only single scenes are involved. On the other hand, tasks that increase the need for endogenous elaboration, for example mentally rotating scenes in the mind’s eye (Lee et al., 2005a) or the phenomenon of boundary extension, where healthy participants extrapolate beyond the view of scenes (Mullally et al., 2012), might be affected in these patients.

Finally, neuroimaging involving healthy controls is also important for examining neural interactions between the hippocampus and vmPFC. For example, it is invaluable for probing the separate functional contributions of the anterior and posterior hippocampus, because in patients damage typically occurs along its entire length. In addition, little is known about the temporal dynamics of autobiographical memory retrieval or scene construction (Fuentemilla et al., 2014, Kaplan et al., 2017). While surface EEG precludes investigation of deep sources such as the hippocampus, techniques like MEG have been shown to detect hippocampal signals (Meyer et al., 2017). This kind of approach would provide traction on examining the directional flow of information during tasks involving autobiographical memory and scene construction (Chen et al., 2008, David et al., 2006), thus providing a robust test of our proposed hierarchical model.

## Conflict of interest

The authors declare no conflicts of interest.
